# Systemic Hypertension Effects on the Ciliary Body and Iris. An Immunofluorescence Study with Aquaporin 1, Aquaporin 4, and Na^+^, K^+^ ATPase in Hypertensive Rats

**DOI:** 10.3390/cells7110210

**Published:** 2018-11-13

**Authors:** Ibrahim González-Marrero, Luis G. Hernández-Abad, Emilia M. Carmona-Calero, Leandro Castañeyra-Ruiz, José A. Abreu-Reyes, Agustín Castañeyra-Perdomo

**Affiliations:** 1 Departamento de Ciencias Médicas Básicas, Facultad de Ciencias de la Salud, Universidad de La Laguna, 38200 La Laguna, Spain; ibra.glez@gmail.com (I.G.-M.); ecarmona@ull.es (E.M.C.-C.); castaney84@gmail.com (L.C.-R.); 2 Instituto de Investigación y Ciencias de Puerto del Rosario, 35600 Puerto del Rosario, Spain; lgarciah@ull.edu.es; 3 Servicio de Oftalmología, Hospital Universitario de Canarias, 38200 La Laguna, Spain; jaabreureyes@gmail.com

**Keywords:** aquaporin 1, aquaporin 4, Na^+^/K^+^ ATPase, iris, ciliary body, hypertension

## Abstract

Aquaporin 1 (AQP1) and aquaporin 4 (AQP4) have been identified in the eye as playing an essential role in the formation of the aqueous humor along with the Na^+^/K^+^ ATPase pump. Different authors have described the relationship between blood pressure, aqueous humor production, and intraocular pressure with different conclusions, with some authors supporting a positive correlation between blood pressure and intraocular pressure while others disagree. The aim of this work was to study the effect of high blood pressure on the proteins involved in the production of aqueous humor in the ciliary body (CB) and iris. For this purpose, we used the eyes of spontaneously hypertensive rats (SHR) and their control Wistar-Kyoto rats (WKY). Immunofluorescence was performed in different eye structures to analyze the effects of hypertension in the expression of AQP1, AQP4, and the Na^+^/K^+^ ATPase α1 and α2 subunits. The results showed an increase in AQP1 and Na^+^/K^+^ ATPase α1 and a decrease in AQP4 and Na^+^/K^+^ ATPase α2 in the CB of SHR, while an increase in AQP4 and no significant differences in AQP1 were found in the iris. Therefore, systemic hypertension produced changes in the proteins implicated in the movement of water in the CB and iris that could influence the production rate of aqueous humor, which would be affected depending on the duration of systemic hypertension.

## 1. Background

The connection of systemic hypertension with changes in intraocular pressure (IOP) have not been clarified to date. High blood pressure (BP) could contribute to increase IOP due to different causes such as overproduction or damage to the outflow of the aqueous humor [1,2]. Many population-based studies have found a strong positive correlation between BP and IOP [3,4,5,6,7,8,9], but not with age**.** According to other authors, there is a strong positive association between systolic arterial pressure (SAP) and IOP, and a weaker association between diastolic arterial pressure (DAP) and IOP [10].

In animal models, it has been reported that eight-month old SHR rats showed a lower IOP (7.8 ± 0.2 mm Hg) than normotensive WKY rats (15.9 ± 0.4 mm Hg) [11,12]. On the other hand, a slight increase of the IOP (18.4 ± 0.8 mmHg) when compared to WKY (15.8 ± 0.9 mmHg) has been observed in eight-week old SHR rats [13].

**Figure 1:**
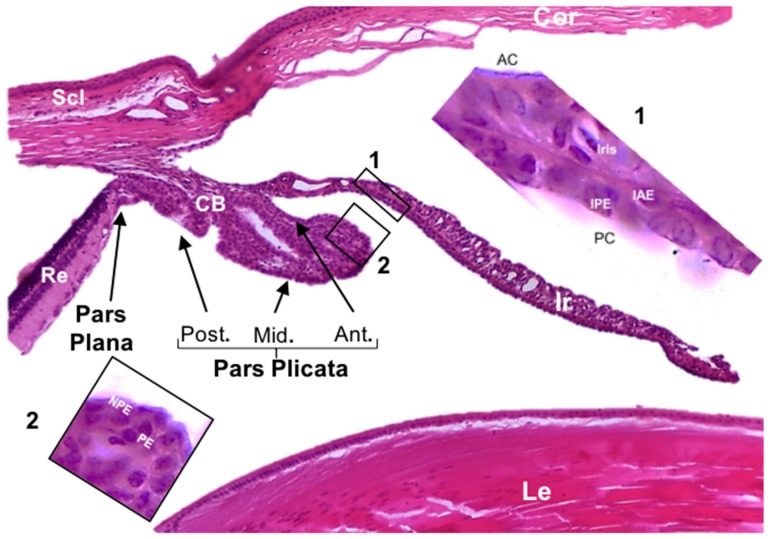
Hematoxylin-eosin stains on the rat eye indicating the ciliary body and iris structures. AC: anterior chamber; CB: ciliary body; Cor: cornea; IAE: iris anterior epithelium; IPE: iris posterior epithelium; Ir: iris; Le: lens; PC: posterior chamber; Re: retina; Scl: sclerotic.

The ciliary epithelium of the ciliary body (CB) and the trabecular meshwork are involved in the production and absorption of aqueous humor in the eye. The ciliary epithelium is composed of pigmented epithelium (PE) and non-pigmented epithelium (NPE) covering the CB (Figure 1), and the tight junctions of NPE form a barrier that separates different compartments and is called the blood-aqueous barrier [14]. The NPE is the forward continuation of the neural layer of the retina and is responsible for the secretion of aqueous humor, while the PE is the forward continuation of the retinal pigment epithelium and is related to solute uptake from the blood [15].

It has been reported that aquaporins (AQP) and Na^+^/K^+^ ATPase control the rate of aqueous humor formation and are located in CB epitheliums and the iris [14]. AQP expression has been investigated in the ocular tissues of humans, mice, rats, dogs, and rabbits [16,17]. AQP studies suggest that AQPs are involved in regulating the water balance in ocular tissues to maintain transparency in the cornea and lens. They also regulate osmolarity, aqueous humor production, and retinal homeostasis. Furthermore, other investigations have implicated AQPs in the etiology of a variety of ocular diseases including retinal and corneal edema, Sjögren’s syndrome, cataracts, and other retinopathies [18,19,20,21]. AQP1 and AQP4 are present in the epithelium of the CB and iris where they facilitate fluid secretion and absorption in the eye and are involved in the regulation of pressure, volume, and tissue hydration [13,22,23].

α1 and α2 subunits of Na^+^/K^+^ ATPase are present in the CB bilayer epithelium. The α1 subunit is present on the basolateral surface of the PE, while the α2 subunit is densely located in the NPE on the side facing the aqueous humor. Furthermore, earlier works have suggested that the Na^+^/K^+^ ATPase α1 in the PE might control overall sodium secretion to the aqueous humor while the Na^+^/K^+^ ATPase α2 may be responsible for the entry of sodium to the ciliary epithelium bilayer across the basolateral surface of the PE, then diffuse via gap junctions to the NPE to exit through the Na^+^/K^+^ ATPase α2 on the NPE cell’s basolateral surface [14,24,25].

Therefore, the aim of this study was to analyze AQP1, AQP4, and Na^+^/K^+^ ATPase expression by immunofluorescence indifferent ocular structures, and to determine whether changes in BP have any influence on the proteins related to aqueous humor formation in hypertensive rats to elucidate whether changes in BP may affect the expression patterns of these proteins.

## 2. Methods

### 2.1. Experimental Animal Protocol

All procedures were carried out following our institutional guidelines for animal experimentation. Wistar-Kyoto rats (WKY) rats and spontaneously hypertensive rats (SHR) were obtained from Charles River Laboratories (Barcelona). Animals were housed in the same environment (25 °C, 12:12-h light-dark cycle) and allowed free access to food and water. All experiments were conducted according to the European Directive 2010/69/EU for the maintenance and use of laboratory animals, which was approved by the Committee of Animal Use for Research at the University of La Laguna. The number of animals used as well as the stress and suffering of these subjects during handling and experimentation were minimized. 

Eyes from 20 male rats sacrificed at 26 weeks of age, subdivided into two groups, were used: (a) ten WKY rats as a control group, and (b) ten SHR rats were fixed by intracardiac perfusion with Bouin’s fluid, dehydrated, and embedded in paraffin under standard conditions. The eyes were cut into four serial sagittal sections. One of the serial sections was stained by the hematoxylin-eosin method. 

All animals were weighed and blood pressure (BP) data were taken before sacrifice at 26 weeks. The systolic BP and diastolic BP were measured by a tail-cuff method with the rats under a conscious condition using a noninvasive BP measurement system (Panlab Non-Invasive Blood Pressure System for Rodents and Dogs. Harvard Apparatus, Cambridge, UK). The rats were placed in plastic restrainers and a cuff with a pneumatic pulse sensor was attached to the tail. The rats were habituated to this procedure for seven days before experiments were performed. BP was taken and determined three times blind to the randomization sequence at each time point and the mean values were used as the result. The same evaluator always took SAP and DAP measurements. The differences between WKY and SHR were significant when a Student’s *t*-test was applied (* *p* < 0.01).

### 2.2. Immunofluorescence 

Tissue sections were prepared for immunofluorescence as previously described [26]. All sections were incubated overnight at 4 °C with an appropriate primary antibody: rabbit anti-AQP4 (ab2218 Sigma-Aldrich, Merck KGaA, Darmstadt, Germany, 1:2500), mouse anti-AQP1 (Ab9566 Abcam, Canbridge, UK 1:1000), mouse anti-Na^+^/K^+^ ATPase α1-subunit (Ab 7671 Abcam, 1:400), and rabbit anti-Na^+^/K^+^ ATPase α2-subunit (07-674 Millipore, Burlington, MA, USA, 1:400). The primary antibodies were validated by Western blot in eye tissue. The procedure for performing immunofluorescence has been previously described [26] and the sections were incubated with the following secondary antibodies: Cyanine 3 (Cy3) dye goat anti-rabbit IgG (Invitrogen, Carlsbad, CA, USA, 1:200) and Alexa Fluor 488 dye goat anti-mouse IgG (Invitrogen, 1:200). Nuclei were stained with 4′-6′ diamidino-2-phenylindole (Invitrogen, 1:5000). After washing, samples were mounted in Vectashield Medium (Vector Laboratories Inc., Orton Southgate, Peterborough, UK) for viewing with a confocal microscope (FV1000 Olympus, Olympus Europa Holding, Hamburg, Germany). The omission of incubation in the primary antibody was used as a negative control. 

### 2.3. Image Acquisition and Immunofluorescence Quantification

Fluorescence intensities from images were analyzed by densitometry. Immunofluorescence slides were converted to digital images by using a confocal microscope FV1000 Olympus as 8-bit acquisitions of color. Image analysis was conducted in ImageJ (v. 1.43 u, NIH, Bethesda, MD, USA). Regions of interest (ROI) were selected and the RGB images were subsequently split into three 8-bit grayscale images containing the red, green, and blue components of the original. The selection of the immunostaining zone was made with the freehand tool of ImageJ and added to the ROI manager. The mean of the obtained values (relative units: r.u.) was calculated and plotted for each mean fluorescence value of the antibodies. A Kolmogorov–Smirnov test was used to check data normality in the statistical analysis of data; all data showed a non-normal distribution. Data were analyzed by the Mann–Whitney *U* test; the statistical analysis was performed with IBM SPSS Statistic 21 software (New York, NY, USA) where data were considered as statistically significant at * *p* < 0.05.

## 3. Results

### 3.1. BP and Body Weight

The mean body weight was 386 ± 2.1 g for the WKY rats and 352 ± 1.9 g for the SHR rats. The mean of the SAP was 131 ± 2.9 mmHg and the DAP was 58 ± 1.7 mmHg in WKY rats; the mean of the SAP was 179 ± 1.7 and the DAP was 64 ± 2.7 in SHR rats (Table 1). 

### 3.2. AQP1 in the Ciliary and Iris Epithelium 

**Figure 2:**
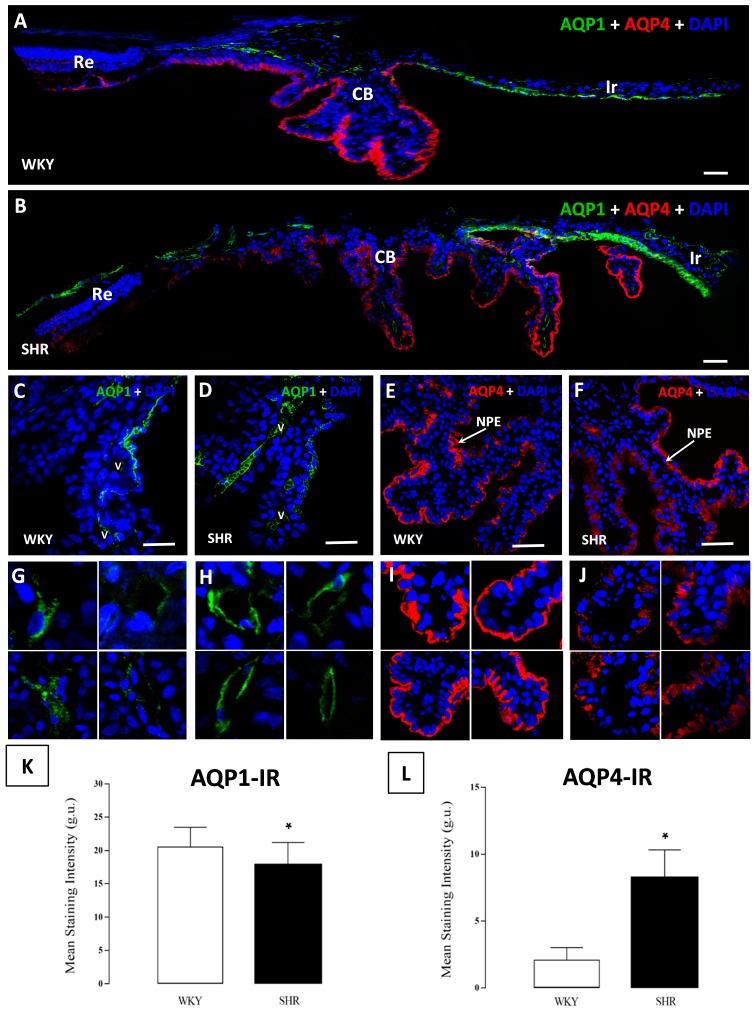
Confocal microscopy images of the expression of AQP1 and AQP4 in the ciliary body of WKY (**A**) rats and SHR (**B**) rats. A reduction of AQP1 was observed in the anterior part of the NPE of the CB in SHR when compared to WKY (**C**,**D**), while in the blood vessels of the CB, an increase of AQP1 was observed in the SHR (**G**,**H**) rats. AQP4 was located in the NPE in the basal membrane (**A**,**B**), with the expression being higher in WKY than in SHR (**E**,**F**). An increase of CB immunofluorescence with AQP4 is shown in (**I**,**J**). At the bottom, values of stain intensities in relative units (r.u.) for AQP1 (**K**) and AQP4 (**L**) are represented as the means ± SD (*n* = ten animals per group) * *p* < 0.05 WKY vs. SHR rats. Ir: iris; CB: ciliary body; Re: retina; NPE: non-pigmented epithelium. Scale bars 40 μm.

**Figure 3:**
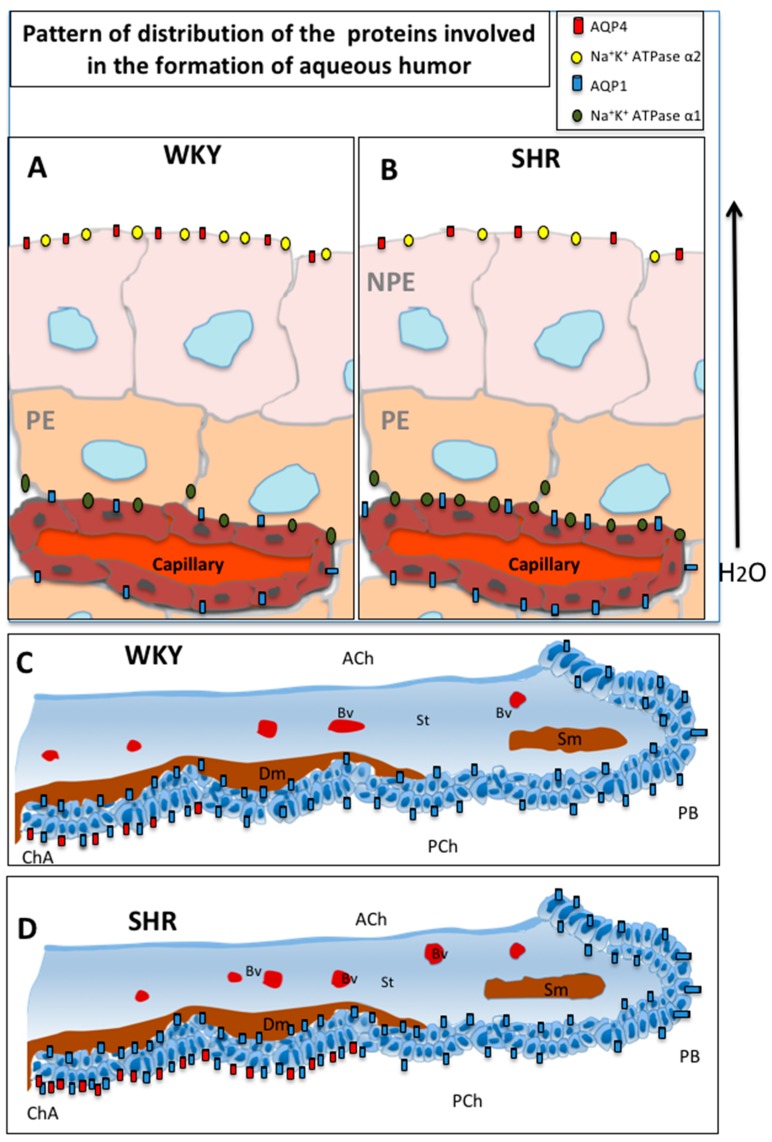
Schematic drawing of the ciliary body (**A**,**B**) and iris (**C**,**D**) showing the distribution of AQP1, AQP4, and Na^+^/K^+^ ATPase in WKY and SHR rats. Ach: anterior chamber; Bv: blood vessel; ChA: chamber angle; Dm: dilator muscle; NPE: non-pigmented epithelium; PCh: Posterior chamber; PE: pigmented epithelium; PD: pupillary border; Sm: sphincter muscle; St: stroma.

The iris and ciliary body were positive for AQP1-immunofluorescence (IF) where only ciliary epithelial cells localized anterior to the pars plicata, endothelial cells of CB blood vessels and iris showed immunofluorescence (Figure 2A,B). In the CB, NPE cells were stained, whereas the PE cells did not show AQP1-IF AQP1 was present in the apical and basolateral membranes in NPE cells of the CB (Figure 2C,D). For all other pars plicata and pars plana, neither NPE cells nor PE cells displayed AQP1-IF. The intensity of AQP1-IF in the ciliary epithelial cells was higher in WKY than SHR rats (Figure 2C,D). A higher expression of AQP1 in the CB was detected in the endothelial cells of the blood vessels and this AQP1-IF was found widely distributed throughout the ciliary body, where immunoreaction was higher in the SHR than in the WKY rats (Figure 2G,H,K and Figure 3).

**Figure 4:**
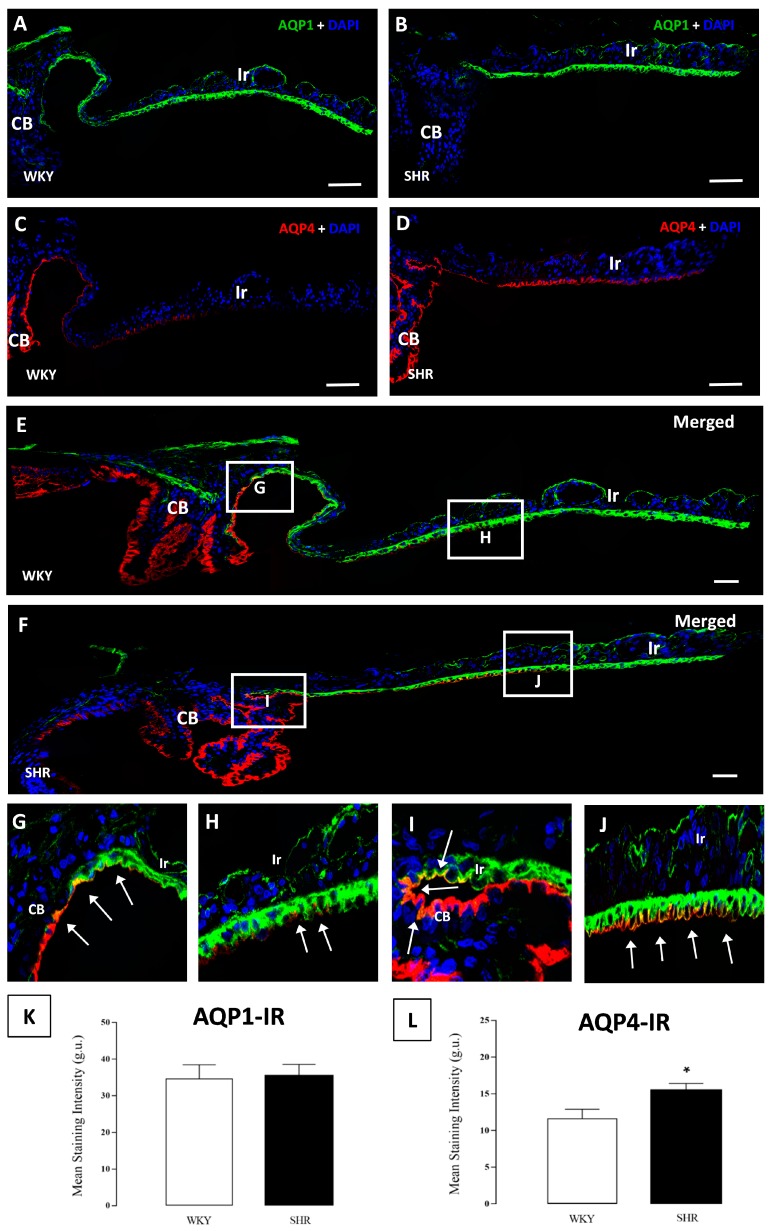
Confocal microscopy images of the expression of AQP1 and AQP4 in the iris of WKY (**E**) rats and SHR (**F**) rats. No differences were found in the iris AQP1 staining between WKY and SHR rats (**A**,**B**). A reduction in AQP4 expression was observed in the epithelium, which went towards the pupillary rim, and was higher in WKY rats (**C**,**D**). Images (**G**,**I**) represent the co-localization of AQP1 and APQ4 in the transition zone between the CB and iris, while (**H**,**J**) represent the co-localization in the iris. At the bottom, values of stain intensities in r.u. for AQP1 (**K**) and AQP4 (**L**) are represented as the means ± SD (*n* = ten animals per group) * *p* < 0.05 WKY vs. SHR rats. Ir: iris; CB: ciliary body. Scale bars 40 μm.

Both layers of epithelial cells of the CB continued into the iris, AQP1–IF was observed in the anterior part of the iris epithelium (AIE) and the posterior part of the iris epithelium (PIE) (Figure 4A,B). Apical and basolateral plasma membranes were stained in the PIE, although AQP1-IF was only present in basal membranes in the AIE, this immunoreaction in the iris was found throughout the epithelium, and no differences in the expression of AQP1 were detected in SHR when compared to WKY rats (Figure 3 and Figure 4A,B,K).

### 3.3. AQP4 in the Ciliary and Iris Epithelium

AQP4-IF was found in both the iris and CB but was detected to a lesser extent in the iris epithelium (Figure 4A,B). An immune reaction to AQP4 was only detected in the basal membrane facing the aqueous humor in the NPE of the CB, which was distributed throughout the pars plicata. The detected expression of AQP4-IF was lower in the SHR than in the WKY rats (Figure 3 and Figure 4E,F,L).

Part of the AQP4 expression was found in the first part of the PIE, but only in the basal membrane, and it was observed that the expression of AQP4 continued in the PIE until it disappeared altogether. In the case of the SHR rats, the AQP4-IF was greater and also spread into more space across the iris (Figure 3 and Figure 4C,D,L).

### 3.4. AQP1 and AQP4 Co-Localization in Ciliary and Iris Epithelium

The presence of AQP4 and AQP1 in the ciliary epithelium and iris has been described above; AQP1 was mainly found in the iris epithelium although it was observed in the most anterior part of pars plicata. On the other hand, AQP4 was mainly expressed in the ciliary body NPE and its expression continued until the iris epithelium. A co-localization of both AQP1 and AQP4 was observed in an area in the junction of both structures (Figure 4E–G,I). Furthermore, there was an increase in both the extension and quantity of AQP4 in the iris of SHR when compared to WKY rats (Figure 3 and Figure 4E,F,H,J).

### 3.5. Na^+^/K^+^ ATPase in the Ciliary Epithelium

**Figure 5:**
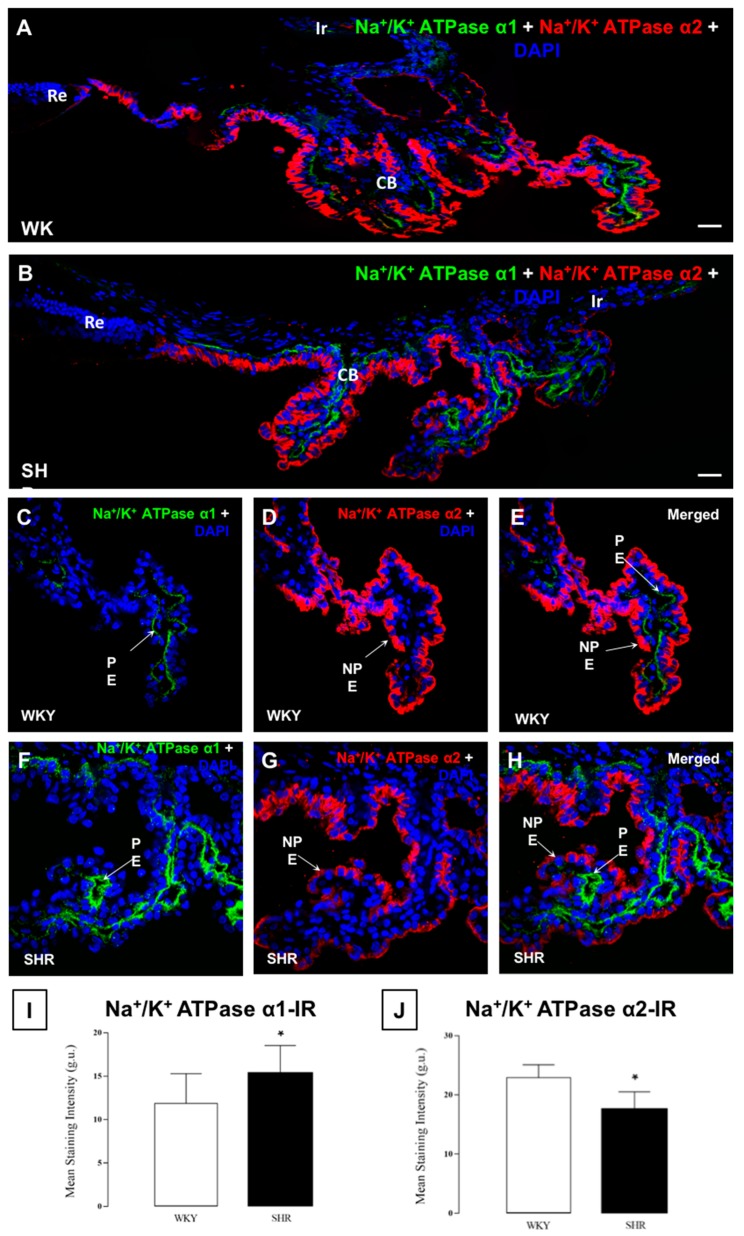
Confocal microscopy images of the expression of Na^+^/K^+^ ATPase α1 and α2 subunits in the ciliary body of WKY (**A**) rats and SHR (**B**) rats. The Na^+^/K^+^ ATPase α1 subunit is located in the basal membrane of the PE of the CB and the staining was higher in SHR when compared to WKY rats (**C**,**F**). A decrease in the expression of α2 in SHR (**D**,**G**) rats was observed in the basal membrane of the NPE of the ciliary body. Images (**E**,**H**) show the localization of both subunits in the CB. At the bottom, values of stain intensities in r.u. for Na^+^/K^+^ ATPase α1 (**I**) and Na^+^/K^+^ ATPase α1 (**J**) are represented as the means ± SD (*n* = ten animals per group) * *p* < 0.05 WKY vs. SHR rats. Ir: iris; CB: ciliary body; Re: retina; NPE: non-pigmented epithelium; Pe: pigmented epithelium. Scale bars 40 μm.

Although α subunits of Na^+^/K^+^ ATPase have the same function, they can be found in different tissues in the eye. The Na^+^/K^+^ ATPase α1 subunit was found in the basolateral membrane of the PE while the Na^+^/K^+^ ATPase α2 subunit was observed on the membrane of the NPE in contact with the aqueous humor of the CB (Figure 5A,B). 

The Na^+/^K^+^ ATPase α1 subunit was found in all pars plicata in the basolateral zone of the PE and surrounding the blood vessels, and its expression was higher in the SHR than in the WKY rats (Figure 5C,F,I). However, the expression of the Na^+^/K^+^ ATPase α2 subunit in the NPE, which was located on the pole facing the aqueous humor, was lower in the SHR than in the WKY rats (Figure 5D,G,J).

## 4. Discussion

The data here regarding the cellular location of the studied proteins agreed with those of other authors [27,28]. The AQP4 was found in the basal membrane of the CB NPE while AQP1 was expressed in the endothelium basal pole of the CB blood vessels. Furthermore, in agreement with [28], AQP1 was also found in both the PIE and AIE of the whole iris, whereas AQP4 was only found in the basal part of the PIE. In the work of Yamaguchi et al. [28], the authors divided the expression of AQP1 and AQP4 into three different regions: one that included most of the iris and expressed only AQP1; another zone where there was co-localization of AQP4 and AQP1 and was situated between the posterior part of the iris and the anterior pars plicata; and finally, an area that included the medial and posterior pars plicata where only AQP4 was expressed. 

The results here showed the same location patterns described above, although differences in the expression of AQP1 and AQP4 with arterial hypertension were found (Figure 3). In addition, the Na^+/^K^+^ ATPase α1 subunit was located in the PE and the Na^+/^K^+^ ATPase α2 subunit was present in the basal membrane of CB NPE in line with Araraki et al. [25].

The correlation between BP and aqueous humor formation and IOP is not clear; several studies have described different and opposite results: one study found no correlation of IOP with SAP values [29], another study hypothesized that the increase in BP could be associated with an elevated IOP, leading to increased risk of glaucoma [30], and another one found that arterial hypertension increased IOP slightly, but had an important negative effect on ocular perfusion [31].

Furthermore, in rats, four weeks of chronic hypertension compromised the benefit of high blood pressure against high IOP. This effect has been partially associated with a reduced capacity for ocular blood flow to autoregulate in response to high IOP in chronic hypertension. Structural changes to blood vessels arising from chronic hypertension may underlie some of our observations, but longer interventions are needed to evoke the long-term components (vascular and neural) of this response [32].

Experimental animals such as SHR showed lower intraocular pressure, despite high arterial blood pressure, than the normotensive WKY. The CB blood vessels in SHR showed a significant dilation of the vessels when compared to WKY. A vascular rarefaction has been reported in the capillaries of the CB as well as in the choroid capillaries [11,12]. The number of fenestrations found in the endothelium of the capillaries in all CB regions was reduced by about half in SHR. In addition, hyalinization of the connective tissue around the vessels in the CB has been observed in SHR. This low IOP described in SHR [11,12] could be explained by the low AQP4 expression described in the results here, which may be considered the cause of the reduced aqueous formation and is also supported by the decrease of the Na^+^/K^+^ ATPase α-2 found here to be clearly low in CB in SHR.

It is possible that in cases of an increase in systemic BP, this would lead to elevated perfusion in the CB, which produces an increase of ultrafiltration and aqueous humor production [33]. However, this increase in aqueous humor formation would not be so evident; compensatory mechanisms may exist when BP elevation has only recently been established and there is still no arterial damage or vascular remodeling. On the other hand, a significant reduction in intraocular pressure has been described in AQP1 knockout (KO) mice, which was attributed more to the reduced production of aqueous humor than output alterations [34].

In this respect, the results obtained in the present study are in line with the above, and AQP1 expression was greater in SHR rats than in WKY rats. In addition, Na^+^/K^+^ ATPase α1, which is involved in the passage of Na^+^ and in creating an osmotic gradient that allows water to pass through, was also greater in the WKY than in the SHR rats. Na^+^/K^+^ ATPase in the CB is modulated by various factors that affect aqueous fluid [35,36].

The above suggests that there is an increase in perfusion in CB. This finding agrees with the results described by a research study in SHR rats, where a slight increase in intraocular pressure (22.5 vs. 20.2 mmHg) was found when compared to controls at eight weeks of age [24]. However, a decrease in AQP4 and a lower expression of the Na^+^/K^+^ ATPase α2 observed in SHR here were responsible for the reduced passage of water to the posterior chamber in the formation of aqueous humor. These findings are consistent with research work on SHR rats that showed a lower IOP (7.8 vs. 15.9 mmHg) than that in WKY rats, and the decrease of IOP developed in parallel to the increase of BP between five and twenty-one weeks of age [12].

According to other authors [37,38], the iris may be involved in the production of aqueous humor. These authors have reported an expression of AQP1 in the iris, both in the PIE and the AIE, and that AQP4 is expressed in the basal membrane of the PIE. Furthermore, it has been suggested that AQP1 expression in the iris epithelium is related to the regulation of aqueous humor volume [28]. It has also been described that the epithelium of the iris is more active in the production of aqueous humor during fetal development than the ciliary body. This pattern contrasts with that in the adult eye, where the ciliary body is the dominant site of AQP expression [38]. In hypertensive rats, AQP1 and AQP4 are greater in the iris and the region of posterior iris–anterior pars plicata, respectively. In addition, in the area where they co-locate, the marking intensity is higher in SHR compared to WKY rats due to the increased extension of AQP4 expression in SHR. If it could be confirmed that the iris participates in the formation of aqueous humor, these results would imply a higher production of aqueous humor as a result of arterial hypertension.

One study reported that hypertensive younger people who were still without vessel damage could benefit from high BP to increase their ocular perfusion pressure, while older people with narrowed vessel lumen may have lower ocular perfusion pressure [39]. It could be assumed from the results here that the increase of AQP1 and Na^+^/K^+^ ATPase α1 in SHR rats would increase the passage of water from the vessels to the CP cells and that there is a decrease in AQP4 and Na^+^/K^+^ ATPase α2 to reduce the production of aqueous humor. This compensatory mechanism could produce a normal reduction in aqueous humor formation for a time, but would fail over time, which would explain the contradictory results reported by the different authors.

## 5. Conclusions

The results reported here suggest that systemic hypertension induces changes in protein expression related to the formation of aqueous humor. These changes are associated with an increase in the ocular perfusion pressure where adaptive mechanisms prevent an elevation of the formation of aqueous humor, thus keeping the IOP constant. The relationship between high BP and high IOP also probably depends on how long the hypertension lasts, in such a way that hypertension could produce a decrease in IOP at the beginning, but long term systemic hypertension produces an increase in IOP. The alterations of the CB, iris, and AQPs produced by high blood pressure may play an important role in this phenomenon. Further studies with older specimens and longer periods of untreated hypertension may be of interest in shedding light on these issues.

## Figures and Tables

**Table 1:** Mean values of body weight, systolic arterial pressure (SAP) and diastolic arterial pressure (DAP) in WKY and SHR rats. The differences between WKY and SHR were significant when a Student’s *t*-test was applied (* *p* < 0.01).

	WKY	SHR
Body weight (g ± SEM)	386 ± 2.1	352 ± 1.9 *
SAP (mmHg ± SEM)	131 ± 2.9	179 ± 1.7 *
DAP (mmHg ± SEM)	58 ± 1.7	64 ± 2.7 *
